# Detection of plant virus particles with a capacitive field-effect sensor

**DOI:** 10.1007/s00216-021-03448-8

**Published:** 2021-07-09

**Authors:** Melanie Jablonski, Arshak Poghossian, Michael Keusgen, Christina Wege, Michael J. Schöning

**Affiliations:** 1 Institute of Nano- and Biotechnologies, FH Aachen, Heinrich-Mußmann-Str. 1, 52428 Jülich, Germany; 2 Institute of Pharmaceutical Chemistry, Philipps University Marburg, Marbacher Weg 6-10, 35032 Marburg, Germany; 3 MicroNanoBio, Liebigstr. 4, 40479 Düsseldorf, Germany; 4 Institute of Biomaterials and Biomolecular Systems, University of Stuttgart, Pfaffenwaldring 57, 70569 Stuttgart, Germany; 5 Institute of Biological Information Processing (IBI-3), Forschungszentrum Jülich GmbH, 52425 Jülich, Germany

**Keywords:** Plant virus, Capacitive field-effect sensor, *Tobacco mosaic virus* (TMV), Label-free detection, Zeta potential

## Abstract

Plant viruses are major contributors to crop losses and induce high economic costs worldwide. For reliable, on-site and early detection of plant viral diseases, portable biosensors are of great interest. In this study, a field-effect SiO_2_-gate electrolyte-insulator-semiconductor (EIS) sensor was utilized for the label-free electrostatic detection of *tobacco mosaic virus* (TMV) particles as a model plant pathogen. The capacitive EIS sensor has been characterized regarding its TMV sensitivity by means of constant-capacitance method. The EIS sensor was able to detect biotinylated TMV particles from a solution with a TMV concentration as low as 0.025 nM. A good correlation between the registered EIS sensor signal and the density of adsorbed TMV particles assessed from scanning electron microscopy images of the SiO_2_-gate chip surface was observed. Additionally, the isoelectric point of the biotinylated TMV particles was determined via zeta potential measurements and the influence of ionic strength of the measurement solution on the TMV-modified EIS sensor signal has been studied.

## Introduction

Harmful organisms, like animal pests, weeds, and plant pathogens, have reduced the productivity of crops since the beginning of agriculture. To avoid such crop losses, farmers have developed various strategies. Nowadays, thanks to modern technologies, crop plants can be protected against a lot of vermin that caused crop loss and famines in the past by using insecticides, fungicides, or genetic modification of the plants [1]. Despite this progress, there are still threats that cannot be averted, especially those induced by viruses. About 15% of worldwide economically important crops are impaired by plant diseases and 30% of plant diseases are induced by plant viruses, leading to economic losses in agriculture of more than 50 billion € per year worldwide [2, 3]. Examples are given by the *zucchini yellow mosaic virus* and *papaya ringspot virus* which cause damaging diseases in cucurbit crops [4], the *barley yellow dwarf virus* that infects cereal crops, and several *cassava mosaic viruses* which have led to a devastating pandemic in east and central Africa [5, 6] (for a good overview, see also [5, 7, 8]). Probably the most famous plant virus is the *tobacco mosaic virus* (TMV), the first discovered plant virus in history and today one of the most studied viruses. It is widely distributed and infects tobacco, tomato, bell pepper, and other members of the family *Solanaceae* as well as plants in about 30 further families, while it is harmless for mammals [9]. The worldwide loss caused by TMV is up to 100 million USD per year [10, 11]. TMV stunts the growth of the infected crop and induces mosaic-like patterns on the leaves of many host species. TMV particles have a nanotube-like structure (length: 300 nm, outer diameter: 18 nm, inner channel: 4 nm). Each TMV nanotube consists of a single-stranded RNA which is embedded between 2130 helically arranged identical coat protein subunits. TMV can spread through the atmosphere and can be transmitted from plant to plant mechanically. It is one of the most stable viruses and can retain its infectivity outside of plant cells in soil or in smoking tobacco for years [9]. During recent years, TMV nanoparticles have been considered increasingly not only as infectious agents, but also as highly attractive nanoscale material for bionanotechnology [12–15] and biochemical sensing applications [16–20].

To develop control strategies for plant viral diseases, reliable and early detection methods are required [3]. Enzyme-linked immunosorbent assays (ELISA) and polymerase chain reaction (PCR)-based methods are most commonly used techniques for the detection of plant disease-causing viruses (including TMV), and there is still ongoing research in this field [21–25]. Immunoassay technologies offer a high specificity and options for on-site detection of plant viruses; however, they have some limitations with regard to low virus concentrations and being one-time sensors. This way, they are usually applied to confirm plant diseases after visual symptoms have already appeared [26, 27]. PCR methods, on the other hand, are very accurate and specific enough to detect very low pathogen concentrations; however, on-site detection with PCR-based methods is hardly realizable [26, 27]. Another approach describes the analysis of plant volatile organic compounds (VOC) as possible biomarkers for plant diseases. Here, the natural variation in the VOC profile within plant species is challenging, because they can mask the changes due to stress and the presence of diseases, and usually lack specificity [28].

To overcome these limitations, portable fast, easy-to use biosensor devices for on-site detection are of great interest. As an example for TMV detection, biosensors based on surface plasmon resonance (SPR) and quartz-crystal microbalance (QCM) have been introduced [29–31]. These sensors rely on molecularly imprinted polymers as receptor layer mimicking the geometrical structure of the nanoparticles to be detected. In contrast, field-effect and bioelectronic devices enable as charge-sensitive devices a direct, label-free electrical monitoring of such particles; above all, they have been widely applied for the development of numerous chemical sensors and biosensors [32–39]. Small size, fast response time, ability for real-time measurements, robustness, and compatibility with micro- and nanofabrication technologies make them attractive to design plant virus-assisted biosensors [19, 20] and to detect charged intact virus particles (see, e.g., recent review [40]), including TMV [41].

Recently, we reported on a highly sensitive, long-term stable penicillin biosensor based on a nanoparticle-modified, capacitive field-effect Al/p-Si/SiO_2_/Ta_2_O_5_ electrolyte-insulator-semiconductor (EIS) structure, where TMV particles loaded on the Ta_2_O_5_ surface serve as nano-scaffolds for an extremely dense immobilization of enzymes (penicillinase) [20]. In addition, these structures have been used for “proof-of-concept” field-effect experiments to detect charged TMV particles adsorbed onto this sensor surface [41]. Here, Ta_2_O_5_ with a nearly Nernstian pH sensitivity (56–58 mV/pH [42, 43]) was selected as gate insulator. On the other hand, it has been reported that due to the interplay between the pH and charge sensitivity of field-effect biosensors, an electrostatic detection of charged molecules by their intrinsic charge can be best achieved on gate insulator surfaces with low pH sensitivity [44, 45]. Therefore, to achieve higher sensitivity to the TMV particle charge in this work, we use a capacitive EIS sensor consisting of an Al/p-Si/SiO_2_ structure having SiO_2_ as gate insulator with sub-Nernstian pH sensitivity (34–38 mV/pH [46, 47]). In addition, the Al/p-Si/SiO_2_ structures can be fabricated more easily and cost-effectively (two fabrication steps, namely, deposition of Ta layer and thermal oxidation to Ta_2_O_5_, were excluded), which represents an important commercial factor for a later large-scale production of on-site, low-cost biosensors.

The SiO_2_-gate EIS sensors are applied for the electrical detection of charged intact TMV particles loaded from solutions with different TMV concentrations ranging from 0.025 nM (0.001 mg/mL) to 5 nM (0.2 mg/mL), whereby a broader concentration range could be studied than in our previous work [41]. The sensitivity of EIS sensors to TMV particles is investigated by means of the constant-capacitance (ConCap) method, while the density of adsorbed TMV particles on the SiO_2_ surface is evaluated from scanning electron microscopy (SEM) images. For a better understanding of the label-free detection of the TMV particles by their intrinsic charge, zeta potential (ZP) measurements for the determination of the isoelectric point (pI) of the biotinylated TMV particles are conducted for the first time. Finally, an influence of the ionic strength (Debye screening effect) of the measurement solution on the TMV-modified EIS sensor signal is studied and discussed for the first time.

## Materials and methods

### Fabrication of EIS sensor chips

To achieve the Al/p-Si/SiO_2_ layer structure, a p-doped silicon wafer (~385 μm thickness, 1–5 Ωcm) was thermally oxidized for 30 min at 1000 °C in O_2_ atmosphere, creating a 30-nm-thick SiO_2_-gate insulator layer. During this oxidation process, an unwanted SiO_2_ layer was also formed on the back side of the p-Si, which was removed by etching with hydrofluoric acid. Then, 300 nm Al was deposited on the rear side of the wafer to provide an electrical contact to the Si. In a final step, this wafer was tempered for 10 min in N_2_ atmosphere at 400 °C. The fabricated wafer was separated to 1 cm × 1 cm chips and cleaned for 5 min in an ultrasonic bath consecutively with acetone, isopropanol, ethanol, and deionized water, respectively.

### Preparation of TMV solutions

As a model plant virus particle, we use a genetically modified TMV variant with a cysteine residue exposed on each coat protein [48], which was already utilized in our previous works for biosensing purposes as nano-scaffold for high-density immobilization of enzymes on a sensor surface [18–20, 41]. Bifunctional maleimide-polyethylenglycol-biotin linkers (EZ-Link Maleimide-PEG11-Biotin (Thermo Scientific, Rockford, IL, USA)) are covalently bound to the thiol groups of these cysteine sites (for details of TMV isolation and biotinylation, see [19, 20, 49]). The TMV stock solution with a concentration of 125 nM (5 mg/mL) in 10 mM sodium-potassium-phosphate buffer (SPP, 10 mM NaH_2_PO_4_ (Merck, Darmstadt, Germany) and 10 mM K_2_HPO_4_ (Carl Roth, Karlsruhe, Germany), pH 7) was stored at 4 °C. For ZP measurements, the TMV stock solution was diluted with 10 mM SPP (15 mM ionic strength) to a concentration of 0.1 mg/mL (2.5 nM) and adjusted to different pH values between pH 7 and pH 3 by titration with 1 M HCl (Sigma-Aldrich, Darmstadt, Germany). For the TMV loading experiments, differently concentrated TMV solutions were prepared by diluting the TMV stock solution with 0.1 M SPP buffer, pH 7, and an ionic strength of 0.15 M.

### Field-effect measurements

The operation principle of EIS sensors is specified in [36], and the mechanism of signal generation induced by the adsorption of TMV particles is discussed in [41]. Summarized, EIS sensors are sensitive to any potential changes that occur at the gate insulator/solution interface. In the case of a p-type semiconductor, as it is used in the present study, the adsorption of negatively charged particles, e.g., TMV particles, reduces the width of the space charge region in the semiconductor, whereby the total capacitance of the sensor structure will increase. By characterization of EIS sensors in the capacitance-voltage (*C–V*) mode, this leads to a shift of the measurement curve to more positive (or less negative) voltages, which can be seen particularly well in the depletion region of the *C–V* curve. In the ConCap measurement mode, which is used in this study, the capacitance in the depletion region of the *C–V* curve (usually ~60% of the maximum capacitance) is set constant by applying a sign-inverted voltage to the reference electrode and, thus, compensating potential changes at the gate surface generated due to the adsorption of TMV particles. This way, the EIS sensor response (output signal) is recorded dynamically over time. Since the presented field-effect sensor detects TMV particles by their intrinsic charge, the ionic strength of the electrolyte solution represents an important parameter. The charge of the virus particles may partially be screened by counter ions. Thus, the ionic strength of the solution has an impact on the sensor behavior and may also affect the TMV adsorption. Due to that, for all electrolyte solutions used in this study, the concentration as well as the ionic strength is specified.

**Fig. 1:**
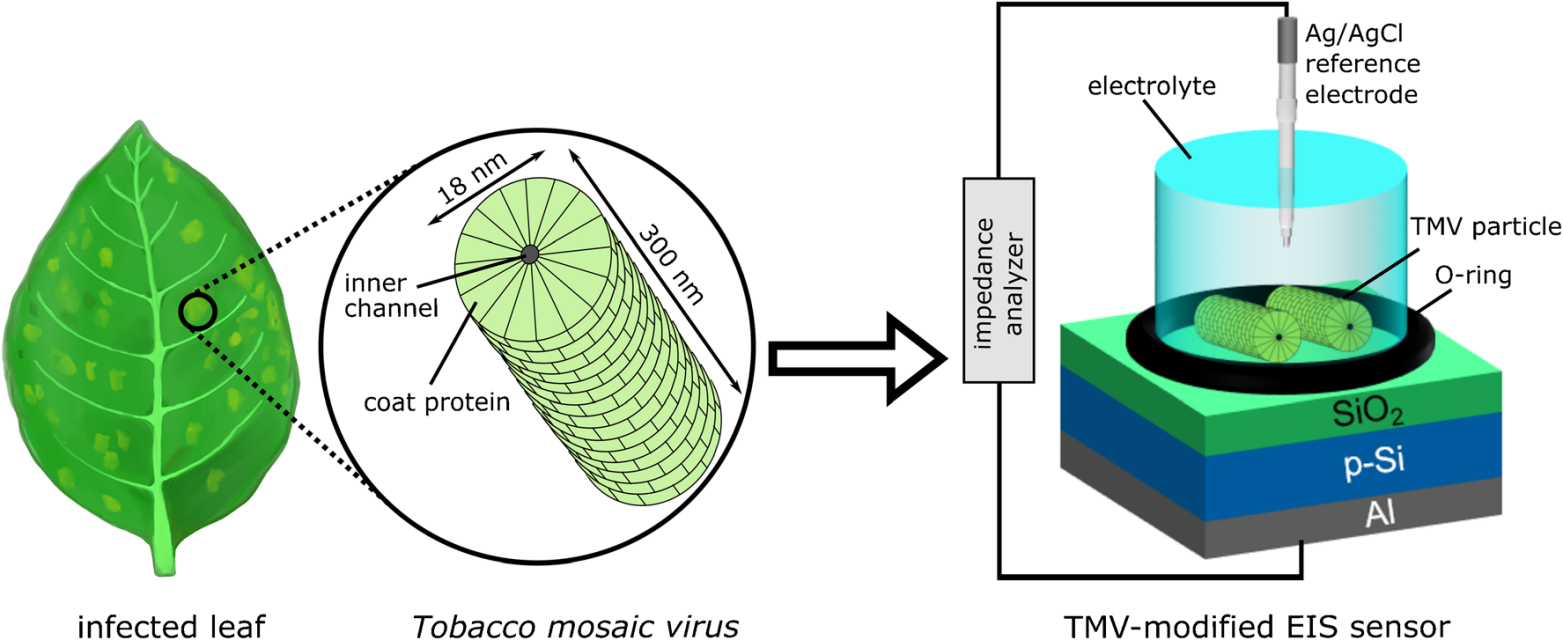
Schematic of a TMV-infected leaf with mosaic-like patterns (left) and a sketch of a TMV nanotube with regularly ordered coat proteins (middle). Schematic measurement setup of a TMV-modified EIS sensor with p-doped silicon, SiO_2_ as gate insulator, and aluminum as rear-side contact (right)

For the detection of TMV particles with SiO_2_ EIS sensors, the EIS chips were mounted in a homemade measurement chamber and sealed with an O-ring, as depicted in Fig. 1. Thereby, 0.5 cm^2^ of the SiO_2_-gate surface was accessible for TMV adsorption and in contact with the electrolyte solution during the electrochemical measurements. The reference electrode (Ag/AgCl, Metrohm, Filderstadt, Germany) was immersed in the measurement solution and connected to an impedance analyzer (Zahner Zennium, Zahner Elektrik, Kronach, Germany). By connecting the Al rear side contact with the impedance analyzer, the electric circuit was closed.

ConCap measurements have been performed in 0.33 mM phosphate-buffered saline (PBS) solution (pH 7) before and after loading the sensor surface with different TMV concentrations; ionic strength was 5 mM. For studying the influence of ionic strength, PBS buffer solutions between 6.5 mM PBS (100 mM ionic strength) and 0.06 mM PBS (1 mM ionic strength) have been used. By diluting a 10-mM PBS stock solution (~154 mM ionic strength) with deionized water, the respective buffer concentrations were achieved. For TMV loading, the sensor chip was incubated with 50 μL of the appropriate TMV solution for 1 h at room temperature in a humid chamber. Afterwards, the sensor was washed three times with 0.33 mM PBS buffer to get rid of non-adsorbed TMV particles.

### pI determination of biotinylated TMV particles by ZP measurements

The working principle of the TMV-modified EIS sensor is based on detecting the intrinsic charge of the virus particle. Therefore, the pI (defined as the pH value at which the electrical net charge of a particle is zero) is an important parameter for this detection method. The pI gives information about the sign of a particle charge depending on the pH value. By that, the expected direction of the signal change, induced by the adsorption of charged TMV particles to the gate surface, can be determined. The pI of native TMV (strain vulgare, U1) is described to be about pH 3.5: TMV particles are negatively charged at pH values above 3.5 and positively charged at pH values below 3.5 [50, 51]. To our best knowledge, there is no information in the literature about the pI of biotinylated TMV particles. Therefore, it was analyzed in the course of this study. As an established method for pI determination, the measurement of the ZP was selected [52]. The ZP represents the electric potential at the slipping plane between the immobile and mobile layers, which is defined by the electrical double layer theory [53]. In this work, ZP analysis was carried out with a Litesizer 500™ Particle Analyzer (Anton Paar GmbH, Graz, Austria) by dynamic light scattering method (DLS). The ZP was examined at a laser wavelength of 660 nm for different pH values between pH 3 and pH 7. For each pH value, three individual measurements were conducted at 25 °C.

### SEM analysis of the TMV-loaded SiO_2_-gate chip surface

For morphological characterization of the TMV-loaded SiO_2_ surface, SEM images were taken from 6 representative areas of the chip surface, using a **JEOL** JSM-7800F Schottky field-emission microscope (JEOL GmbH, Freising, Germany). Before imaging, a ca. 3–5 nm Pt/Pd film was sputtered onto the chip surface. The density of adsorbed TMV particles on the sensor surface was determined from the SEM images using the image evaluation software ImageJ Fiji (Rasband, W.S., U.S. National Institutes of Health, Bethesda, MD, USA, https://imagej.nih.gov/ij/). For calculating the TMV density, the TMV particle was defined as a 300-nm long nanotube. Though, TMV particles can adsorb as long end-to-end structures, or as smaller particle fractions with lengths often in the range of 50–200 nm. Therefore, the number of the differently sized particles was multiplied with corresponding factors.

## Results and discussion

### ZP measurements

**Fig. 2:**
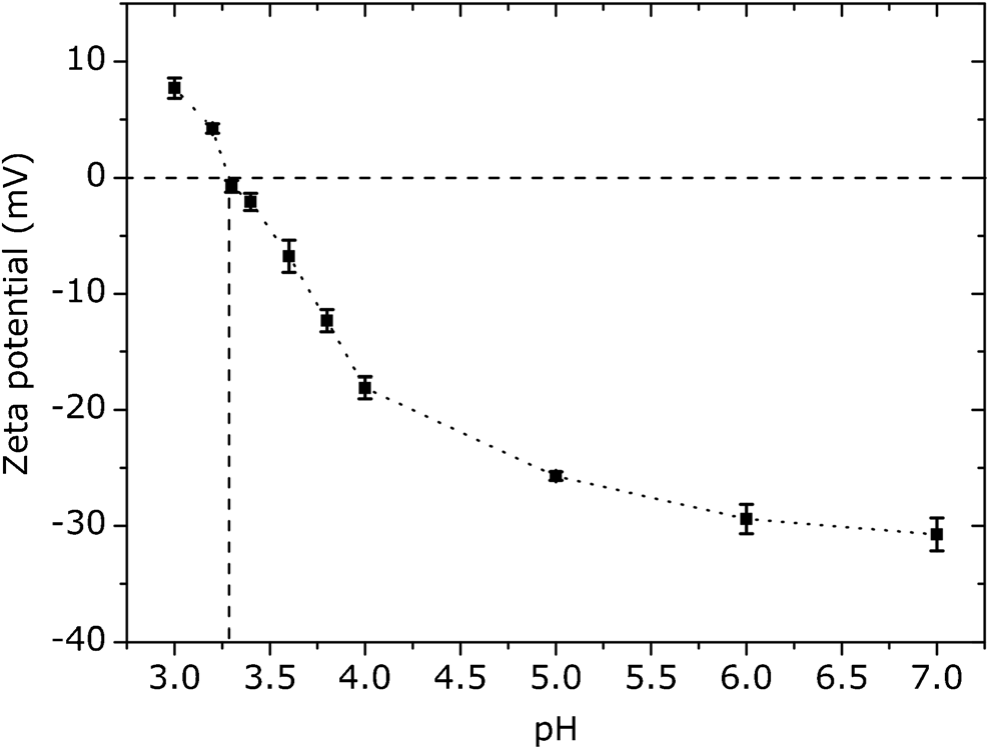
ZP of biotinylated TMV at different pH values between pH 3 and pH 7. Measurements have been performed with 2.5 nM TMV in 10 mM SPP buffer at 25 °C

For ZP measurements, the biotinylated TMV particles were dissolved in 10 mM SPP buffer, with a TMV concentration of 2.5 nM. The ZP was measured at different pH values between pH 3 and pH 7. Figure 2 represents the mean ZP values of three individual measurements with varying pH values. For the lowest value at pH 3, the ZP was 7.7 mV, while it reached −30.7 mV for pH 7. Note that the pI is defined by the crossing point of the measured ZP with the 0-mV axis.

As indicated in Fig. 2, the pI of biotinylated TMV particles is determined to be pH 3.3, showing that it is in good agreement with the pI reported for native TMV particles [50, 51]. It should be mentioned that the ZP is influenced by many parameters, such as ionic strength, ion composition, or temperature, which underlines that an exact comparison with literature data is only possible when having the same measurement conditions. Nevertheless, our results confirm that the biotinylated TMV particles were negatively charged at pH 7. Consequently, a shift of the measurement signal to more positive voltage values is expected during the ConCap measurements, when TMV particles are adsorbing to the SiO_2_-gate surface.

### Label-free detection of TMV particles with SiO_2_-gate EIS sensors

**Fig. 3:**
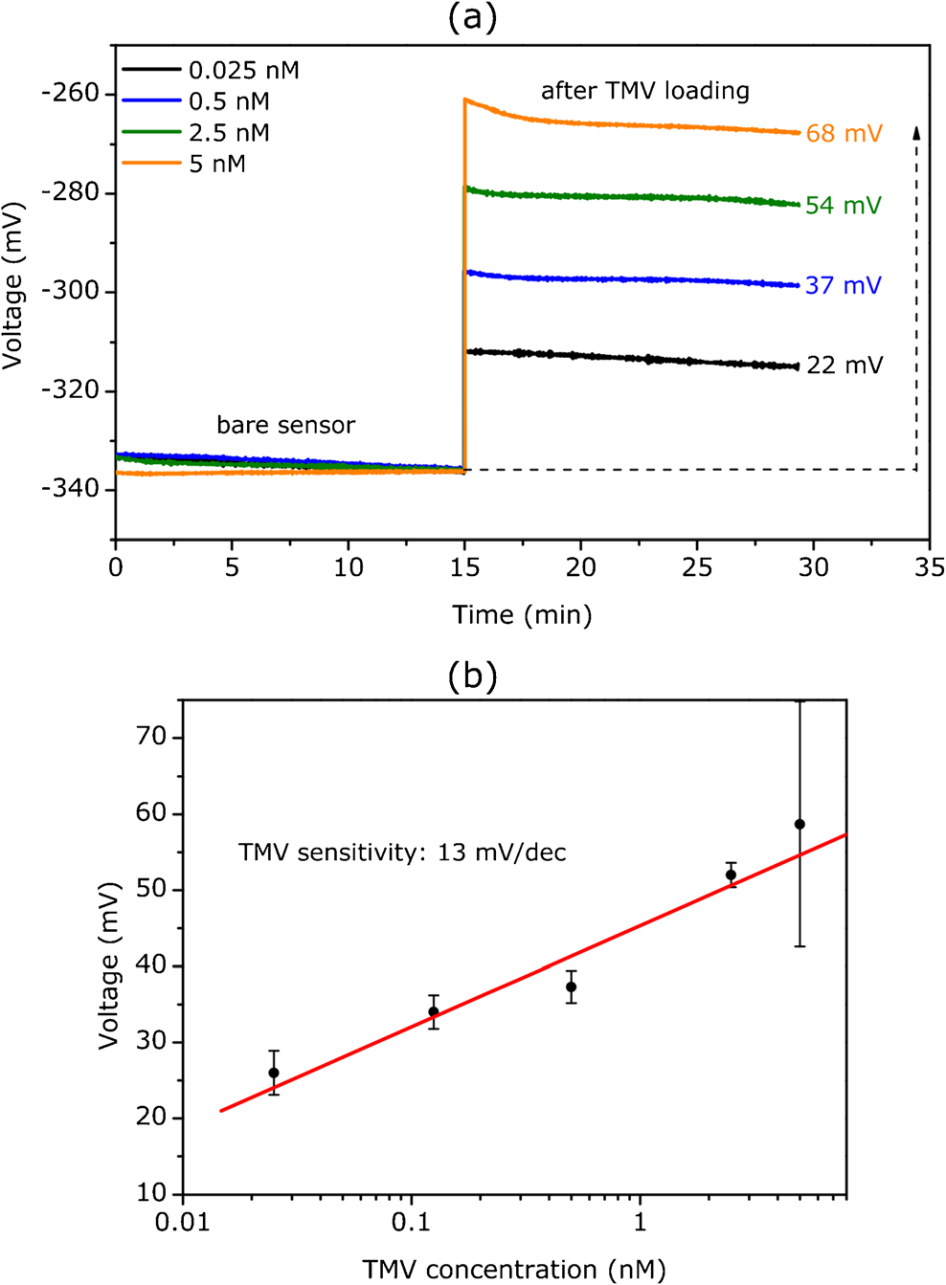
(a) Exemplary ConCap curves recorded in 0.33 mM PBS buffer (pH 7, ionic strength: 5 mM) before and after loading of TMV from differently concentrated solutions between 0.025 nM and 5 nM TMV. The ConCap curves are normalized on the measurement curve recorded at a TMV concentration of 0.025 nM. (b) Mean calibration curve (n = 3) evaluated from the respective ConCap measurements

Since EIS sensors are charge-sensitive devices and because biotinylated TMV particles are negatively charged at pH > 3.3, it can be expected that loading of TMV particles on the SiO_2_ surface will alter the gate surface charge. To evaluate the TMV sensitivity of the SiO_2_ EIS sensor, ConCap measurements have been performed in 0.33 mM PBS buffer (pH 7) before and after loading of virus particles on the sensor surface from differently concentrated TMV solutions. For each TMV concentration, three SiO_2_-gate EIS chips were characterized. In Fig. 3a, exemplary ConCap curves can be seen, which were registered before (bare sensor) and after loading of different TMV concentrations between 0.025 nM (0.001 mg/mL) and 5 nM (0.2 mg/mL). As expected, the ConCap signal of the EIS sensor modified with negatively charged TMV particles is shifted in the direction of less negative (or more positive) gate voltages. In the ConCap mode, the feedback control circuit applies a more positive voltage on the gate via the reference electrode to compensate gate potential changes caused by the negative charge of the adsorbed TMV particles, to keep the total EIS capacitance constant. Thus, the direction of the ConCap signal shift serves as an indicator for the successful loading of TMV particles, while the amplitude of the signal change could give information about the amount of the adsorbed virus particles.

With increasing TMV concentration from 0.025 to 5 nM, the signal change rises from 22 to 68 mV, evidencing that the charge at the sensor surface becomes more negative as a consequence of an increased number of adsorbed TMV particles. It is worth to note that even at the lowest TMV concentration of 0.025 nM used in this work, the sensor shows a sufficiently detectable sensor signal.

Figure 3b depicts the mean calibration curve evaluated from three ConCap measurements per TMV concentration conducted with three individual sensors. The mean TMV sensitivity of SiO_2_-gate EIS sensors was 13 ± 1.4 mV/dec in the range of 0.025–5 nM. For a TMV concentration of 5 nM, a high standard deviation for the sensor signal can be seen. The reason for this is that the surface density of adsorbed TMV particles on one of the three sensors was significantly lower, which has been supported by scanning electron microscopy examination. Consequently, this particular sensor showed a clearly lower signal amplitude resulting in a high standard deviation. This means that the reproducibility of SiO_2_ surface treatment procedure and/or TMV adsorption process has to be further improved. At the same time, this underlines that the sensing principle is working properly, since the sensor signal correlates with the number of adsorbed TMV particles on the gate insulator. Concluding, the results displayed in Fig. 3 demonstrate the great potential of capacitive field-effect EIS sensors for the label-free detection of intact TMV particles, especially considering the fact how simple the sensor platform is.

We have found only a few articles related to the detection of intact TMV particles with biosensors. Dickert et al. reported a linear detection range from 100 ng/mL to 1000 μg/mL by combining molecular imprinting technique and QCM [29]. In the work of Boltovets et al., no information about the TMV concentrations is given, making a comparison with the achieved results difficult [30]. Dubs et al. used an antibody-based SPR sensor but employed only one TMV concentration of 100 μg/μL [31].

Since field-effect EIS sensors detect charge changes on the gate surface induced by the adsorbed TMV particles, the ConCap signal changes in Fig. 3a should correlate with the number or density of adsorbed TMV particles. Therefore, the density of the adsorbed TMV particles on the SiO_2_-gate surface was studied by SEM. SEM images were taken from the surfaces of the same EIS chips, which were already used for ConCap measurements.

**Fig. 4:**
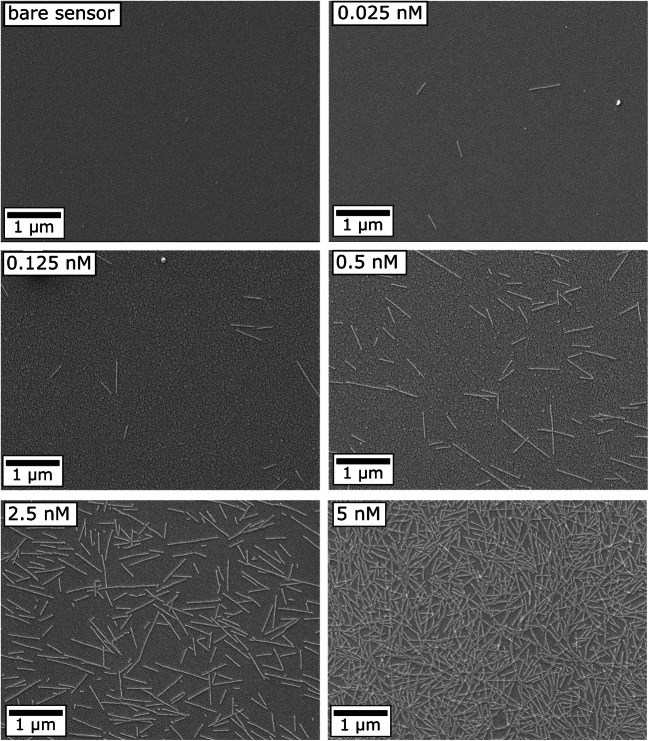
Exemplary SEM images of the SiO_2_-gate sensor surface before (bare sensor) and after loading of virus particles from differently concentrated TMV solutions between 0.025 and 5 nM. The SEM images were taken after ConCap measurements with the same sensors

In Fig. 4, exemplary SEM images of a bare EIS sensor and EIS sensors loaded with different TMV concentrations between 0.025 and 5 nM are displayed. For all concentrations, the TMV particles are relatively homogeneously distributed with some particles present as longer end-to-end aggregated structures or as shorter fragments. An increasing number of adsorbed TMV particles with higher TMV concentration in solution can be clearly seen. The calculated mean density of adsorbed TMV particles raised from (0.014 ± 0.009) × 10^9^ TMV/cm^2^ to (4.2 ± 2.4) × 10^9^ TMV/cm^2^ with increasing the TMV concentration in solution from 0.025 to 5 nM, respectively.

**Fig. 5:**
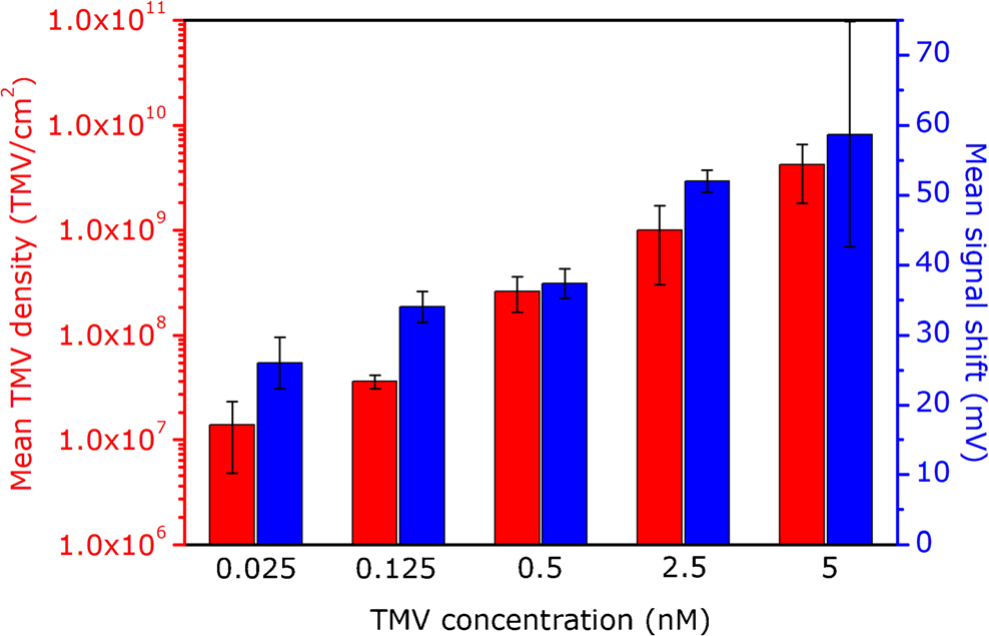
Bar chart representing mean TMV densities evaluated from three individual sensors after TMV loading from differently concentrated TMV solutions between 0.025 and 5 nM (red). Mean signal shifts measured with the same sensors after TMV loading (blue)

The bar chart in Fig. 5 summarizes the dependencies of the mean density of TMV particles and mean signal shifts (both evaluated for three EIS sensors) on the concentration of TMV particles in solution. As expected, the mean sensor signal shifts correlate well with the mean TMV densities. The higher the density of loaded virus particles, the larger are the recorded ConCap signals induced by the adsorbed charged particles. These results demonstrate that the developed sensor could be applied to estimate/quantify the number or density of virus particles adsorbed on various gate materials, without the utilization of a complex and costly SEM equipment.

### Influence of ionic strength on the sensor signal

It is known that electrolyte-gated field-effect devices, including the capacitive EIS sensors studied in this work, are generally capable to detect potential/charge changes that occur at the gate surface or within the order of the Debye length from the surface [37, 54–56]. The Debye length is defined as the distance over which the electrostatic potential decays by the factor of 1/*e*, and is inversely proportional to the ionic strength of the electrolyte solution. Due to the screening of the virus charge by counter ions in the surrounding solution, the EIS sensor signal could be affected significantly by the Debye length/ionic strength and by the distance between the virus charge and the gate surface.

**Fig. 6:**
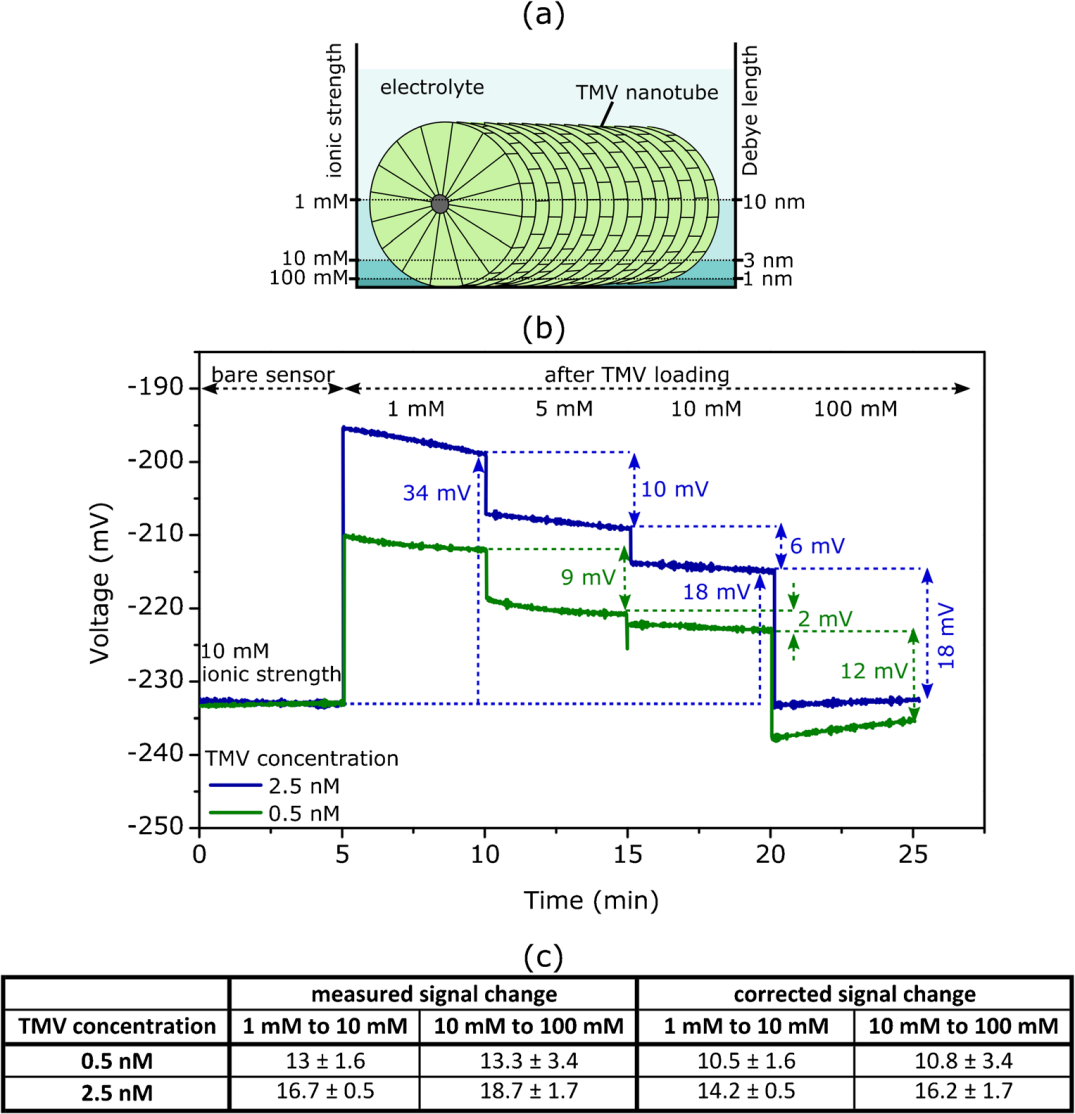
(a) Schematic illustration of a TMV nanotube on the sensor surface and Debye length in surrounding electrolyte with different ionic strengths. (b) ConCap curves of SiO_2_-gate EIS sensors after TMV loading from differently concentrated TMV solutions (0.5 nM TMV (green) and 2.5 nM TMV (blue)) measured in PBS buffer solution (pH 7) with different ionic strengths between 1 and 100 mM, respectively. (c) Mean signal changes after changing the ionic strength from 1 to 10 mM and from 10 to 100 mM, respectively. The corrected signal changes were obtained taking into account the Na^+^ -ion sensitivity of the SiO_2_ layer, reported in the literature (~2.5 mV/pNa [46])

In Fig. 6a, a schematic illustration of a TMV nanotube on the sensor surface and the Debye length in the surrounding electrolyte with different ionic strengths is shown. Due to the nanotube-like structure of TMV particles with an outer diameter of 18 nm, the TMV charge is not confined directly to the sensor surface, but it is distributed through some distance away from the surface. With increasing ionic strength of the electrolyte, the fraction of TMV charge, which remains in the double layer and thus will be mirrored in the EIS sensor signal, is decreased. For example, in an electrolyte solution with an ionic strength of 100 mM and a corresponding Debye length of ~1 nm, most of the TMV particle charge will be at a distance greater than the Debye length from the surface and, therefore, cannot be detected with the field-effect EIS sensor.

Therefore, to find out the impact of the charge screening effect, the ConCap signal of TMV-modified SiO_2_-gate EIS sensors was recorded in PBS buffer (pH 7) with different ionic strengths of 1 mM, 5 mM, 10 mM, and 100 mM (the calculated values of the Debye length were ca. 10 nm, 4.3 nm, 3 nm, and 1 nm, respectively). Typical ConCap curves of EIS sensors with TMV particles loaded from the solution with two different TMV concentrations of 0.5 nM and 2.5 nM are depicted in Fig. 6b. For both TMV concentrations, it is visible that with increasing ionic strength of the solution (1 to 100 mM), the sensor signal is shifted in the direction of more negative voltages, which corresponds to less negatively charged TMV particles due to the more efficient screening of the TMV charge by counter ions. As expected, the amplitude of the ConCap signal of TMV-loaded EIS sensors decreases with increasing the ionic strength. For instance, in the case of the EIS sensor with TMV particles loaded from the solution with a TMV concentration of 2.5 nM, the amplitude of the ConCap signal decreases from 34 to 18 mV by increasing the ionic strength from 1 to 10 mM, respectively. Therefore, to generally achieve a high sensor signal for label-free electrostatic detection of virus particles with field-effect EIS sensors, low ionic strength solution should be chosen. Note, in the “Label-free detection of TMV particles with SiO_2_-gate EIS sensors” section, for example, 5 mM was adjusted.

Figure 6c lists the mean signal changes by different ionic strengths of the measurement solution evaluated from the respective ConCap curves for three individual EIS sensors and for two TMV concentrations. Because the change in the ionic strength of the PBS buffer implicates also a change in the NaCl (buffer component) concentration and since SiO_2_ layers typically have some sensitivity to Na^+^ ions [46, 57], the measured mean signal changes were corrected. The corrected values of signal changes were obtained by taking into account a Na^+^-ion sensitivity for SiO_2_ of ~2.5 mV/pNa reported in [46].

The results achieved in this experiment underline the significant influence of the ionic strength on the performance of virus-modified EIS biosensors, which should be considered in future studies on the application of EIS sensors for plant pathogen detection.

## Conclusion

Plant viruses are a major cause of crop losses worldwide, leading to enormous economic damage. The best strategy to prevent great crop failures is the early detection of virus infections. Due to numerous advantages, like small size, robustness, fast response time, and compatibility with micro- and nanofabrication technologies, electrolyte-gated field-effect devices are very attractive for on-site, real-time, and label-free detection of plant viruses by their intrinsic charge. In this study, a capacitive field-effect SiO_2_-gate EIS sensor for the detection of TMV particles as model pathogen is presented. Since the working principle is based on the electric charge of the TMV particles, first, the pI of the utilized biotinylated TMV particles was determined by means of ZP measurements and shown to be in the range of natural virus strains. The capacitive EIS sensors were characterized by ConCap measurements before and after loading of negatively charged TMV particles of different concentrations on the SiO_2_-gate surface. The mean TMV sensitivity of SiO_2_-gate EIS sensors was 13 ± 1.4 mV/dec in the concentration range of 0.025–5 nM TMV.

The morphology of the TMV-loaded SiO_2_ surface was additionally studied by means of SEM images, from which the density of TMV particles was evaluated. The calculated mean surface density of adsorbed TMV particles rose from (0.014 ± 0.009) × 10^9^ TMV/cm^2^ to (4.2 ± 2.4) × 10^9^ TMV/cm^2^ with increasing the TMV concentration in solution from 0.025 to 5 nM, respectively. Moreover, the ConCap signal shifts correlate well with the density of TMV particles adsorbed on the gate surface: The higher the density of loaded virus particles, the larger was the amplitude of the ConCap signal. In addition, to find out the impact of the charge screening effect, the TMV-modified SiO_2_-gate EIS sensors were characterized in PBS buffers with different ionic strengths between 1 and 100 mM. As expected, due to the more efficient screening of the TMV charge by counter ions, the amplitude of the ConCap signal of TMV-loaded EIS sensors decreased with increasing the ionic strength. The results achieved in this experiment prove the considerable influence of ionic strength on the performance of virus-modified EIS sensors.

Even though the presented sensor shows a high potential for label-free detection of viral particles, the measurements have only been performed in model analytes so far. In real sample analytes, non-specific adsorption of other charged species may generate false-positive signals or mask the usable signal from the target virus particles of interest. The specificity of EIS sensors and the ability to discriminate the virus type are very important aspects and will be studied in further experiments. As for other kinds of field-effect sensors, specificity can be achieved by means of functionalization of the gate surface with receptor molecules, like antibodies specific to the coat protein of the TMV particle, antibody fragments, aptamers, etc. To reduce/eliminate non-specific adsorption, various strategies such as the use of selectively active blocking agents, pre-purifying the biological liquids, on-chip filtering, separation, desalting, pre-concentration, and other strategies have been discussed in the literature, which, in principle, could also be applicable for EIS sensors. As TMV typically accumulates to high concentrations in infected cells, the prospects for a relatively simple selective enrichment protocol from plant raw material are good, but will need careful comparative evaluation.

In conclusion, although the study demonstrates the potential of EIS sensors for label-free detection of intact TMV particles (as a model plant pathogen), this approach could most likely be extended to other plant viruses, too. Capacitive EIS sensors represent an attractive platform for future application as sensors for in-field and real-time, early detection of plant diseases.

## References

[1] Oerke E-C. Crop losses to pests. J Agric Sci. 2006;144:31–43. 10.1017/S0021859605005708.

[2] Islam W, Qasim M, Ali N, Tayyab M, Chen S, Wang L. Management of tobacco mosaic virus through natural metabolites. Rec. Nat. Prod. 2018;12:403–415. 10.25135/rnp.49.17.10.178.

[3] Pallás V, Sánchez-Navarro JA, James D. Recent advances on the multiplex molecular detection of plant viruses and viroids. Front. Microbiol.2018;9:2087. 10.3389/fmicb.2018.02087. 10.3389/fmicb.2018.02087 PMC6139301 30250456

[4] Coutts BA, Kehoe MA, Jones RAC. Minimising losses caused by zucchini yellow mosaic virus in vegetable cucurbit crops in tropical, sub-tropicaland Mediterranean environments through cultural methods and host resistance. Virus Res. 2011;159:141–160. 10.1016/j.virusres.2011.04.015. 10.1016/j.virusres.2011.04.015 21549770

[5] Strange RN, Scott PR. Plant disease: a threat to global food security. Annu. Rev. Phytopathol. 2005;43:83–116. 10.1146/annurev.phyto.43.113004.133839. 10.1146/annurev.phyto.43.113004.133839 16078878

[6] Rey C, Vanderschuren H. Cassava mosaic and brown streak diseases: current perspectives and beyond. Annu. Rev. Virol. 2017;4:429–452. 10.1146/annurev-virology-101416-041913. 10.1146/annurev-virology-101416-041913 28645239

[7] Murray GM, Brennan JP. Estimating disease losses to the Australian wheat industry. Australas. Plant Pathol. 2009;38:558–570. 10.1071/AP09053.

[8] Rybicki EP. A top ten list for economically important plant viruses. Arch. Virol. 2015;160:17–20. 10.1007/s00705-014-2295-9. 10.1007/s00705-014-2295-9 25430908

[9] Peng J, Song K, Zhu H, Kong W, Liu F, Shen T, et al. ast detection of tobacco mosaic virus infected tobacco using laser-induced breakdownspectroscopy. Sci. Rep. 2017;7:44551. 10.1038/srep44551. 10.1038/srep44551 PMC5353609 28300144

[10] Fu Y, Liu D, Zeng H, Ren X, Song B, Hu D, et al. New chalcone derivatives: synthesis, antiviral activity and mechanism of action. RSC Adv.2020;10:24483–24490. 10.1039/D0RA03684F. 10.1039/d0ra03684f PMC9055036 35516226

[11] Chen YH, Guo DS, Lu MH, Yue JY, Li Y, Shang CM, et al. Inhibitory effect of osthole from cnidium monnieri on tobaccomosaic virus (TMV) infection in Nicotiana glutinosa. Molecules. 2020;25:65. 10.3390/molecules25010065. 10.3390/molecules25010065 PMC6982833 31878172

[12] Fan XZ, Pomerantseva E, Gnerlich M. Tobacco mosaic virus: a biological building block for micro/nano/biosystems. J. Vac. Sci. Technol.2013;31:050815. 10.1116/1.4816584.

[13] Love AJ, Makarov V, Yaminsky I, Kalinina NO, Taliansky ME. The use of tobacco mosaic virus and cowpea mosaic virus for the production ofnovel metal nanomaterials. Virology 2014;449:133–139. 10.1016/j.virol.2013.11.002. 10.1016/j.virol.2013.11.002 24418546

[14] Wege C, Koch C. From stars to stripes: RNA-directed shaping of plant viral protein templates –structural synthetic virology for smart biohybridnanostructures. Wiley Interdiscip. Rev. Nanomed. Nanobiotechnol. 2020;12:e1591. 10.1002/wnan.1591. 10.1002/wnan.1591 31631528

[15] Lomonossoff GP, Wege C. Chapter six - TMV particles: the journey from fundamental studies to bionanotechnology applications. In: Palukaitis P.,Roossinck, M. J., editors. Advances in virus research. Academic Press; 2018;102:149–176. 10.1016/bs.aivir.2018.06.003. 10.1016/bs.aivir.2018.06.003 PMC7112118 30266172

[16] Fan XZ, Naves L, Siwak NP, Brown A, Culver J, Ghodssi R. Integration of genetically modified virus-like-particles with an optical resonator forselective bio-detection. Nanotechnology 2015;26:205501. 10.1088/0957-4484/26/20/205501. 10.1088/0957-4484/26/20/205501 25915182

[17] Zang F, Gerasopoulos K, Brown AD, Culver JN, Ghodssi R. Capillary microfluidics-assembled virus-like particle bionanoreceptor interfaces for label-free biosensing. ACS Appl. Mater. Interfaces 2017;9:8471–8479.. 10.1021/acsami.6b14045. 10.1021/acsami.6b14045 28211673

[18] Bäcker M, Koch C, Geiger F, Eber F, Gliemann H, Poghossian A, et al. Tobacco mosaic virus as enzyme nanocarrier forelectrochemical biosensors. Sens. Actuators B 2017;238:716–722. 10.1016/j.snb.2016.07.096.

[19] Koch C, Poghossian A, Schöning MJ, Wege C. Penicillin detection by tobacco mosaic virus-assisted colorimetric biosensors. Nanotheranostics2018;2:184–196. 10.7150/ntno.22114. 10.7150/ntno.22114 PMC5865271 29577021

[20] Poghossian A, Jablonski M, Koch C, Bronder TS, Rolka D, Wege C, et al. Field-effect biosensor using virus particles as scaffolds forenzyme immobilization. Biosens. Bioelectron. 2018;110:168–174. 10.1016/j.bios.2018.03.036. 10.1016/j.bios.2018.03.036 29609165

[21] Yang JG, Wang FL, Chen DX, Shen LL, Qian YM, Liang ZY, et al. Development of a one-step immunocapture real-time RTPCRassay for detection of tobacco mosaic virus in soil. Sensors. 2012;12:16685–16694. 10.3390/s121216685. 10.3390/s121216685 PMC3571805 23211755

[22] Iftikhar Y, Jackson R, Neuman BW (2015). Detection of tobacco mosaic tobamovirus in cigarettes through RT-PCR. Pak J Agric Sci.

[23] Kumar S, Udaya Shankar AC, Nayaka SC, Lund OS, Prakash HS. Detection of tobacco mosaic virus and tomato mosaic virus in pepper and tomatoby multiplex RT–PCR. Lett. Appl. Microbiol. 2011;53:359–363. 10.1111/j.1472-765X.2011.03117.x. 10.1111/j.1472-765X.2011.03117.x 21740446

[24] Kumar S, Prakash HS. Detection of tobacco mosaic virus and tomato mosaic virus in pepper seeds by enzyme linked immunosorbent assay (ELISA).Arch. Phytopathol. Plant Prot. 2016;49:59–63. 10.1080/03235408.2012.658991.

[25] Rettcher S, Jungk F, Kühn C, Krause HJ, Nölke G, Commandeur U, et al. Simple and portable magnetic immunoassayfor rapid detection and sensitive quantification of plant viruses. Appl. Environ. Microbiol. 2015;81:3039–3048. 10.1128/AEM.03667-14. 10.1128/AEM.03667-14 PMC4393444 25710366

[26] Fang Y, Ramasamy RP. Current and prospective methods for plant disease detection. Biosensors. 2015;5:537–5. 10.3390/bios5030537. 10.3390/bios5030537 PMC4600171 26287253

[27] Khater M, de la Escosura-Muñiz A, Merkoçi A. Biosensors for plant pathogen detection. Biosens. Bioelectron. 2017;93:72–86. 10.1016/j.bios.2016.09.091. 10.1016/j.bios.2016.09.091 27818053

[28] Sankaran S, Mishra A, Ehsani R, Davis C. A review of advanced techniques for detecting plant diseases. Comput. Electron. Agric. 2010;72:1–13 10.1016/j.compag.2010.02.007.

[29] Dickert FL, Hayden O, Bindeus R, Mann KJ, Blaas D, Waigmann E. Bioimprinted QCM sensors for virus detection—screening of plant sap. Anal.Bioanal. Chem. 2004;378:1929–1934. 10.1007/s00216-004-2521-5. 10.1007/s00216-004-2521-5 14985911

[30] Boltovets PM, Boyko VR, Kostikov IY, Dyachenko NS, Snopok BA, Shirshov YM. Simple method for plant virus detection: effect of antibodyimmobilization technique. J. Virol. Methods. 2002;105:141–146. 10.1016/S0166-0934(02)00098-8. 10.1016/s0166-0934(02)00098-8 12176151

[31] Dubs MC, Altschuh D, Van Regenmortel MH. Interaction between viruses and monoclonal antibodies studied by surface plasmon resonance.Immunology Letters. 1992;31:59–64. 10.1016/0165-2478(92)90011-C. 10.1016/0165-2478(92)90011-c 1372280

[32] Gramberg B, Kintzios S, Schmidt U, Mewis I, Ulrichs C. A basic approach towards the development of bioelectric bacterial biosensors for the detectionof plant viruses. J. Phytopathol. 2012;160:106–111. 10.1111/j.1439-0434.2011.01867.x.

[33] Gao A, Chen S, Wang Y, Li T. Silicon nanowire field-effect-transistor-based biosensor for biomedical applications. Sens. Mater. 2018;30:1619–1628; 10.18494/SAM.2018.1829.

[34] Wu C, Poghossian A, Bronder TS, Schöning MJ. Sensing of double-stranded DNA molecules by their intrinsic molecular charge using the lightaddressablepotentiometric sensor. Sens. Actuators B 2016;229:506–512. 10.1016/j.snb.2016.02.004.

[35] Pullano SA, Critello CD, Mahbub I, Tasneem NT, Shamsir S, Islam SK, et al. EGFET-based sensors for bioanalyticalapplications: a review. Sensors 2018;18:4042. 10.3390/s18114042. 10.3390/s18114042 PMC6263563 30463318

[36] Poghossian A, Schöning MJ. Capacitive field-effect chemical sensors and biosensors: A status report. Sensors. 2020;20:5639. 10.3390/s20195639. 10.3390/s20195639 PMC7584023 33023133

[37] Kaisti M. Detection principles of biological and chemical FET sensors. Biosens. Bioelectron. 2017;98:437–448.. 10.1016/j.bios.2017.07.010. 10.1016/j.bios.2017.07.010 28711826

[38] Katz E, Poghossian A, Schöning MJ. Enzyme-based logic gates and circuits: analytical applications and interfacing with electronics. Anal. Bioanal.Chem. 2017;409:81–94. 10.1007/s00216-016-0079-7. 10.1007/s00216-016-0079-7 27900435

[39] Yoshinobu T, Miyamoto K, Werner CF, Poghossian A, Wagner T, Schöning MJ. Light-addressable potentiometric sensors for quantitative spatialimaging of chemical species. Annu. Rev. Anal. Chem. 2017;10:225–246. 10.1146/annurev-anchem-061516-045158. 10.1146/annurev-anchem-061516-045158 28375701

[40] Poghossian A, Jablonski M, Molinnus D, Wege C, Schöning MJ. Field-effect sensors for virus detection: From Ebola to SARS-CoV-2 and plant viralenhancers. Front. Plant Sci. 2020;11:598103. 10.3389/fpls.2020.598103. 10.3389/fpls.2020.598103 PMC7732584 33329662

[41] Jablonski M, Poghossian A, Severins R, Keusgen M, Wege C, Schöning MJ. Capacitive field-effect biosensor studying adsorption of tobacco mosaicvirus particles. Micromachines. 2021;12:57. 10.3390/mi12010057. 10.3390/mi12010057 PMC7825068 33418949

[42] Poghossian A, Malzahn K, Abouzar MH, Mehndiratta P, Katz E, Schöning MJ. Integration of biomolecular logic gates with field-effect transducers.Electrochim. Acta 2011;56:9661–9665. 10.1016/j.electacta.2011.01.102.

[43] Chen M, Jin Y, Qua X, Jin Q, Zhao J. Electrochemical impedance spectroscopy study of Ta2O5 based EIOS pH sensors in acid environment. Sens.Actuators B 2014;192:399–405. 10.1016/j.snb.2013.10.129.

[44] Landheer D, Aers G, McKinnon WR, Deen MJ, Ranuarez JC. Model for the field effect from layers of biological macromolecules on the gates ofmetal-oxide-semiconductor transistors. J. Appl. Phys. 2005;98:044701. 10.1063/1.2008354.

[45] Wunderlich BK, Neff PA, Bausch AR. Mechanism and sensitivity of the intrinsic charge detection of biomolecular interactions by field effect devices.Appl. Phys. Lett. 2007;91:083904. 10.1063/1.2775040.

[46] Uslu F, Ingebrandt S, Mayer D, Böcker-Meffert S, Odenthal M, Offenhäusser A. Labelfree fully electronic nucleic acid detection system based on afield-effect transistor device. Biosens. Bioelectron. 2004;19:1723–1731. 10.1016/j.bios.2004.01.019. 10.1016/j.bios.2004.01.019 15142607

[47] Poghossian A, Bäcker M, Mayer D, Schöning MJ. Gating capacitive field-effect sensors by the charge of nanoparticle/molecule hybrids. Nanoscale2015;7:1023–1031. 10.1039/C4NR05987E. 10.1039/c4nr05987e 25470772

[48] Geiger FC, Eber FJ, Eiben S, Mueller A, Jeske H, Spatz JP, et al. TMV nanorods with programmed longitudinal domains of differentlyaddressable coat proteins. Nanoscale. 2013;5:3808–3816. 10.1039/C3NR33724C. 10.1039/c3nr33724c 23519401

[49] Koch C, Wabbel K, Eber FJ, Krolla-Sidenstein P, Azucena C, Gliemann H, et al. Modified TMV particles as beneficialscaffolds to present sensor enzymes. Front. Plant Sci. 2015;6:1137. 10.3389/fpls.2015.01137. 10.3389/fpls.2015.01137 PMC4689848 26734040

[50] Tiu BDB, Kernan DL, Tiu SB, Wen AM, Zheng Y, Pokorski JK, et al. Electrostatic layer-by-layer construction offibrous TMV biofilms. Nanoscale. 2017;9:1580–1590. 10.1039/C6NR06266K. 10.1039/c6nr06266k 28070572

[51] Alonso JM, Gorzny MŁ, Bittner AM. The physics of tobacco mosaic virus and virus-based devices in biotechnology. Trends Biotechnol. 2013;31:530–538. 10.1016/j.tibtech.2013.05.013. 10.1016/j.tibtech.2013.05.013 23849673

[52] Bhattacharjee S. DLS and zeta potential–what they are and what they are not? J. Controlled Release. 2016;235:337–351. 10.1016/j.jconrel.2016.06.017. 10.1016/j.jconrel.2016.06.017 27297779

[53] Greben K, Li P, Mayer D, Offenhäusser A, Wördenweber R. Immobilization and surface functionalization of gold nanoparticles monitored via streamingcurrent/potential measurements. J. Phys. Chem. B. 2015;119:5988–5994. 10.1021/acs.jpcb.5b02615. 10.1021/acs.jpcb.5b02615 25905436

[54] Nishio Y, Uno S, Nakazato K. Three-dimensional simulation of DNA sensing by ion-sensitive field-effect transistor: optimization of DNA position andorientation. Jpn. J. Appl. Phys. 2013;52:04CL01. 10.7567/jjap.52.04cl01.

[55] Lowe BM, Sun K, Zeimpekis I, Skylaris C-K, Green NG. Field-effect sensors - from pH sensing to biosensing: sensitivity enhancement usingstreptavidin-biotin as a model system. Analyst 2017;142:4173–4200. 10.1039/C7AN00455A. 10.1039/c7an00455a 29072718

[56] Poghossian A, Schöning MJ. Label-free sensing of biomolecules with field-effect devices for clinical applications. Electroanalysis 2014;26:1197–1213. 10.1002/elan.201400073.

[57] Schöning MJ, Abouzar MH, Poghossian A. pH and ion sensitivity of a field-effect EIS (electrolyte-insulator-semiconductor) sensor covered withpolyelectrolyte multilayers, J. Solid State Electrochem. 2009;13:115–122. 10.1007/s10008-008-0589-0.

